# Characterization of the murine orthotopic adamantinomatous craniopharyngioma PDX model by MRI in correlation with histology

**DOI:** 10.1371/journal.pone.0197895

**Published:** 2018-05-24

**Authors:** Annett Hölsken, Marc Schwarz, Clarissa Gillmann, Christina Pfister, Michael Uder, Arnd Doerfler, Michael Buchfelder, Sven Schlaffer, Rudolf Fahlbusch, Rolf Buslei, Tobias Bäuerle

**Affiliations:** 1 Institute of Neuropathology, University Hospital Erlangen, Friedrich-Alexander-University Erlangen-Nürnberg, Erlangen, Germany; 2 Institute of Radiology, University Hospital Erlangen, Friedrich-Alexander-University Erlangen-Nürnberg, Erlangen, Germany; 3 Department of Neuroradiology, University Hospital Erlangen, Friedrich-Alexander-University Erlangen-Nürnberg, Erlangen, Germany; 4 Institute of Neurosurgery, University Hospital Erlangen, Friedrich-Alexander-Universität Erlangen-Nürnberg, Erlangen, Germany; 5 International Neuroscience Institute (INI) Hannover, Hannover, Germany; 6 Department of Pathology, Sozialstiftung Bamberg, Bamberg, Germany; George Washington University, UNITED STATES

## Abstract

**Purpose:**

Adamantinomatous craniopharyngiomas (ACP) as benign sellar brain tumors are challenging to treat. In order to develop robust *in vivo* drug testing methodology, the murine orthotopic craniopharyngioma model (PDX) was characterized by magnetic resonance imaging (MRI) and histology in xenografts from three patients (ACP1-3).

**Methods:**

In ACP PDX, multiparametric MRI was conducted to assess morphologic characteristics such as contrast-enhancing tumor volume (CETV) as well as functional parameters from dynamic contrast-enhanced MRI (DCE-MRI) and diffusion-weighted imaging (DWI) including area-under-the-curve (AUC), peak enhancement (PE), time-to-peak (TTP) and apparent diffusion coefficient (ADC). These MRI parameters evaluated in 27 ACP PDX were correlated to histological features and percentage of vital tumor cell content.

**Results:**

Qualitative analysis of MRI and histology from PDX revealed a similar phenotype as seen in patients, although the MRI appearance in mice resulted in a more solid tumor growth than in humans. CETV were significantly higher in ACP2 xenografts relative to ACP1 and ACP3 which correspond to respective average vitality of 41%, <10% and 26% determined histologically. Importantly, CETV prove tumor growth of ACP2 PDX as it significantly increases in longitudinal follow-up of 110 days. Furthermore, xenografts from ACP2 revealed a significantly higher AUC, PE and TTP in comparison to ACP3, and significantly increased ADC relative to ACP1 and ACP3 respectively. Overall, DCE-MRI and DWI can be used to distinguish vital from non-vital grafts, when using a cut off value of 15% for vital tumor cell content.

**Conclusions:**

MRI enables the assessment of craniopharyngioma PDX vitality *in vivo* as validated histologically.

## Introduction

Adamantinomatous craniopharyngiomas (ACP) are histologically benign epithelial tumors of the sellar region arising from embryonic remnants of Rathke’s pouch [1]. The most common ACP variant differs from the second papillary subtype in regards to age distribution, genetic alterations, transcriptomic and epigenomic profiles [2] and histology [3]. ACP are the most common non-neuroepithelial intracerebral neoplasms in children, accounting for 5–11% of intracranial tumors in this age group. A second peak occurs in adulthood between 50–74 years [1]. They are characterized by activating mutations in the beta-catenin encoding gene *CTNNB1* [4, 5].

Morphologically, ACP tumor masses often include multicystic areas harboring a fluid component (which can be described best as “machinery oil-like”) as well as solid parts including nodules and calcifications. On MRI, ACP appear lobulated with cystic T2 hyperintense regions and solid components that vividly enhance after the administration of an intravenous contrast agent on T1 weighted images [6, 7]. Calcifications are common for these tumors which are primarily identified by hyperdense lesions in CT [8]. ACP tend to increase in size and thus may result in the compression of adjacent tissue and structures of the suprasellar region [9].

Histological hallmarks of ACP are the formation of differentiated epithelium arranged in cords, lobules, nodular whorls and irregular trabeculae bordered by palisaded columnar epithelium. Islands of densely packed cells are surrounded by loose aggregates of stellate cells. In these vital tumor structures often non-vital components of pale nodules containing anucleate “ghost cells”/”wet keratin”, large areas of regressive changes i.e. hemosiderin deposits, cholesterol clefts, multinucleated foreign-body giant cells, inflammatory cells and calcifications are found. Cystic cavities comprising cell debris and fibrosis are lined by flattened epithelium [10]. Further characteristic micro morphological features based on vital ACP tumor cells are the formation of expanding finger-like protrusions [11] inducing a tumor-specific cellular environment within the compromised brain tissue [12]. In addition to functional impairment of the pituitary gland, ACP affect adjacent vital structures, such as the optic chiasm, the pituitary stalk and the hypothalamus due to their predominantly suprasellar occurrence and enlargement. This can lead to visual and serious endocrinological disturbances e.g., diabetes insipidus, hyperphagia and obesity as a result of hypothalamus impairment accompanied by significant long-term morbidity and mortality rates [13, 14].

These issues highlight that, despite ACP being classified as benign tumors, their clinical behavior can be aggressive and treatment is challenging. Surgery and radiotherapy are current treatment options, which may be limited if complete tumor resection is not possible or recurrence after radiation treatment occurs. Effective chemotherapeutic options are currently not available. However, recent findings provided a significant insight into the molecular pathogenesis associated with new pharmacological treatment targets and indicated promising radiochemotherapeutic strategies [15–17]. The preclinical testing of these targets *in vivo* makes suitable models indispensable [18]. Beside transgenic mouse models, the best conformance in relation to the human ACP was achieved using the intracranial xenograft model established from human tumor tissue [19, 20, 21]. The engrafted tumors may reproduce the complex 3D morphology of human ACP including all aforementioned non-vital (e.g. calcification and wet keratin) as well as vital structures playing an important role in perpetuation of molecular characteristics [20]. This includes amongst others the activation of the WNT and EGFR signaling pathway with accompanied gene regulatory capacities, which vary between patients. [11, 17, 18, 20]. Thus, the PDX model provides a tool for identifying efficient patient specific treatment in the future [18]. For this purpose, it is of fundamental importance (i) to ensure a sufficient content of vulnerable (vital) tumor proportions before starting extensive treatment testing and (ii) to identify treatment associated changes in a longer course of time due to the relatively slow growth of ACP. To achieve this goal, a non invasive and robust imaging methodology such as MRI is required.

Therefore, in this study we characterized the murine ACP PDX model after the orthotopic implantation of tissue originating from three different patients (ACP1-3) into nude mice (n = 27) performing multiparametric MRI for correlation with corresponding histological appearance and to define conclusive parameters with regard to tumor constitution.

## Materials and methods

### Ethics approval and consent to participate

All experimental protocols using human samples were approved by the Ethical Committee of the University of Erlangen-Nürnberg. Informed consent was obtained from all individual participants included in the study. All methods used were carried out in accordance with the approved guidelines of the Ethical Committee of the University of Erlangen-Nürnberg and in accordance with the Declaration of Helsinki. A declaration of consent for further scientific investigation is available from each patient for all specimens as prescribed by the local ethics committee of the Friedrich-Alexander-University Erlangen-Nürnberg (FAU).

All animal experiments were performed in accordance with the Protection of Animals Act and with the approval of the local Animal Protection Committee: Regierung von Unterfranken in Würzburg (file number 55.2–2532.1-25/14).

### Animal care and tumor induction

The patient derived ACP xenotransplants (PDX) were established as previously described in detail by Stache et al. [20] in five-week-old female NMRI- Fox1^nu^/Fox1^nu^ mice (nude mice) purchased from Janvier Elevage (Le Genest St. Ile, France). An accommodation time upon arrival of at least one week was met to adapt all animals to the new environment prior to the beginning of experimental procedures. All animals are housed in the local center for animal experiments (Franz-Penzoldt-Zentrum, University Hospital Erlangen). The center maintains standardized housing conditions as well as hygiene management according to the guidelines of the European Federation for Laboratory Animal Science Associations (FELASA). The animals are kept under special conditions e.g. pathogen-free using autoclaved cages ventilated with filtered air, a temperature of 25°C, a humidity of 65% as well as a 12/12 hours light-dark cycle. These constant conditions were maintained utilizing an enclosed airflow cabinet (Ehret Bioscape, Uni Protect, Emmendingen, Germany). Furthermore, all animals had free access to sterilized, acidified drinking water (pH 2.5–2.8) and gamma-irradiated feed ad libitum. The environment was enriched with shelters (igloos).

In our study, surgically removed native ACP specimens of three different patients, two males ACP1 and ACP3 (9 and 56 y/o) and one female (ACP2; 28 y/o; Table 1) were used for xenotransplantation. For this purpose, mice were anesthetized with 0.12 mg/g body weight Ketamine in combination with 0.016 mg/g body weight Xylazine by intraperitoneal injection.

**Table 1:** Patient-specific origin of xenografts and study results.

Clinical data				Murine PDX								
Primary tumor	gender	age	CTNNB1 mutation	PDX	Estimated vital tumor cells within the lesion (%)	Average vitality	T2 –Tumor Volume (mm^3^)	CETV (mm^3^)	ADC (10-^3^mm^2^/s)	PE (AU) (from AUC)	AUC (AU)	TTP (s)
**ACP1**	m	9	codon 33 TCT(Ser)>TTT(Phe)	M1	<10	<10%	0.775	0.391	289.96	nap	nap	nap
				M2	<10		0.398	0.528	304.00	nap	nap	nap
				M3	<10		2.051	1.809	580.02	nap	nap	nap
				M4	<10		0.1889	0.000	592.76	nap	nap	nap
				M5	<10		0.143	0.138	nap	nap	nap	nap
				M6	<10		0.189	0.000	197.88	nap	nap	nap
				M7	≤15		0.389	0.394	542.72	nap	nap	nap
**ACP2**	f	28	codon 37 TCT(Ser)>TGT(Cys)	M1	18	41%	3.690	3.639	636.32	3.243	1857.544	688.67
				M2	65		2.267	0.915	823.02	1.241	413.112	688.68
				M3	45		3.397	3.335	653.87	4.368	2894.490	328.32
				M4	35		4.319	4.689	649.76	3.168	2002.002	711.70
				M5	50		4.419	4.662	868.63	4.169	2680.921	633.62
				M6	25		3.239	3.842	955.21	5.035	3243.652	547.53
				M7	35		2.155	2.435	701.18	2.651	1665.557	633.62
				M8	25		4.238	3.806	755.256	2.536	1640.649	484.47
				M9	70		3.783	3.678	707.828	2.038	1363.598	163.15
				M10	45		2.101	2.351	653.96	2.174	1448.397	328.32
**ACP3**	m	56	codon 33 TCT(Ser)>TGT(Cys)	M1	35	~26%	0.563	0.000	431.93	0.801	447.167	203.19
				M2	<10		0.257	0.1381	nap	0.326	165.496	38.030
				M3	15		nap	0.000	559.18	0.405	93.178	7.000
				M4	20		1.510	1.532	843.64	1.567	988.460	273.260
				M5	18		1.520	1.392	588.49	1.863	1058.330	70.060
				M6	<10		0.928	0.379	622.00	0.717	315.002	46.040
				M7	55		1.905	1.428	838.65	0.573	296.725	7.000
				M8	45		0.405	0.107	404.053	0.625	255.027	23.010
				M9	45		1.313	0.475	284.00	0.660	462.662	688.680
				M10	<10		nap	0.000	547.56	1.631	569.779	22.010

During the transplantation procedure the head of the mouse was fixed in a stereotactical frame (Bilaney Consultants, Düsseldorf, Germany). A burr hole with a diameter of 1 mm was drilled 3 mm lateral to the bregma. Through this hole, tumor tissue from surgical specimens was transplanted. We aimed to transplant 1–8 mm^3^ of solid tumor tissue with a sterile cannula. Afterwards, the skin was closed with a suture (ETHILON*II 4–0, Ethicon, Norderstedt, Germany). For pain relief, the animals received 1 mg/g Novaminsulfon (Ratiopharm, Ulm, Germany) with drinking water for three consecutive days. Health condition and behavior monitoring of all animals was performed daily. Body weight was also assessed daily for one week post-operatively, thereafter once weekly. Animals exhibiting the human endpoint criteria defined as weight loss of 20% or atypical behavioral changes were euthanized. For euthanasia, mice were anesthetized with isofluorane and sacrificed by decapitation.

In total, 27 mice received patient derived ACP tumor tissue which is exemplified in Table 1. Seven mice were transplanted with tumor tissue obtained from ACP1 (ACP1M1-7). Tumor tissue from patients ACP2 and ACP3 was transplanted in ten mice respectively (ACP2M1-10 and ACP3M1-10). The clinical data of ACP patients are summarized in Table 1. CTNNB1 mutation status of primary tumor tissue was evaluated as previously described [2].

The patient-specific origin of xenograft models is also shown in Table 1.

PDX in NMRI^nu/nu^ mice originated from native primary tumor tissue of three different patients (ACP 1–3). Clinical data of patients, including age at the time of surgery, gender and CTNNB1 mutation status are shown. The engrafted tumors (ACP1 n = 7; ACP2 n = 10, ACP3 n = 10) of each individual mouse, as well as average vitality, are specified in more detail with regard to vital tumor content reviewing tumors of histologically processed mouse brains. The MRI data determined prior to the scarification of mice include T2 tumor volume, CETV (contrast-enhancing tumor volume), ADC (apparent diffusion coefficient), PE (peak enhancement), AUC (area-under-the-curve) and TTP (time-to-peak). Abbreviations f (female), m (male), nap (no analysis possible)

### Magnetic resonance imaging (MRI)

To provide a direct comparison of tumor appearance in MRI with histology, in this study we used MRI data obtained from PDX mice prior to their immolation and subsequent histological processing of the brain. The period of time after tumor induction via transplantation and the last MRI ranges between 41 and 190 days. In order to elucidate the important question whether the engrafted tumors were growing, we compared CETV (T1 (CM)) and T2 assessed tumor volume 28 days post transplantation (dpt) with tumor volume measured 138 dpt. These data were available from five ACP2 derived xenotransplanted mice (n = 5, S1 Table).

Prior to and during the MRI examination, isoflurane was used in a concentration of up to 1.5% mixed with 20% oxygen to anesthetize the animals. The mice were catheterized through the tail vein to enable contrast-agent application during the measurement. The MRI contrast agent (Gadovist [Bayer Vital GmbH, Leverkusen, Germany]) was applied intravenously with 0.1 mMol/kg bodyweight.

The animals were examined using a small-animal MRI system (ClinScan 70/30, Bruker BioSpin MRI GmbH, Ettlingen, Germany) equipped with a dedicated mouse brain coil and an animal monitoring system. Respiratory rate and body temperature were kept constant.

The MRI protocol (total acquisition time: 42 minutes) consisted of the following sequences: T2 (TR: 2000ms, TE: 42ms, Flip Angle: 180°, Bandwidth: 130Hz/Px, Pixel Size: 0.074x0.074mm^2^, Acquisition Matrix: 252x512, Field of View: 24x38mm, Slice Thickness: 0.7mm), T1 before and after contrast agent application (TR: 500ms, TE: 9ms, Flip Angle: 90°, Bandwidth: 205Hz/Px, Pixel Size: 0.043x0.043mm^2^, Acquisition Matrix: 448x512, Field of View: 19x22mm, Slice Thickness: 0.7mm), diffusion-weighted imaging (DWI) (TR: 8000ms, TE: 60ms, Flip Angle: 90°, Bandwidth: 1860Hz/Px, Pixel Size: 0.172x0.172mm^2^, Acquisition Matrix: 112x128, Field of View: 57x66mm, Slice Thickness: 1.0mm) and dynamic contrast-enhanced (DCE) imaging (TR: 3.9ms, TE: 0.88ms, Flip Angle: 25°, Bandwidth: 490Hz/Px, Pixel Size: 0.172x0.172mm^2^, Acquisition Matrix: 104x128, Field of View: 17x22mm, Slice Thickness: 0.2mm).

For qualitative evaluation of imaging features in humans and mice, MRI in mice (after xenografting) were compared to MRI data from the respective patients ACP 1–3 (before surgical craniopharyngeoma resection) using T1w and T2w images.

#### Parameter calculation

DCE-MRI parameters Area-under-the-curve (AUC), peak enhancement (PE) and time-to-peak (TTP) were calculated using open-source software, Horos (Version 1.1.7, available at (https://www.horosproject.org), in combination with a freely available DCE-plugin (Version 2.3, available at http://kyungs.bol.ucla.edu/software/DCE_tool/DCE_tool.html). T2 and post-contrast T1 weighted images were used to manually segment the tumor volume using Horos (see above) as described before [22]. Using T2 weighted images, hyperintense tumor areas were segmented and on post contrast T1 weighted images, the contrast-enhancing tumor volume (CETV) was determined. MRI operating software (Syngo, Version VB15, Siemens Healthcare, Erlangen, Germany) calculated and provided apparent diffusion coefficient (ADC)-maps that served as source to readout ADC-values using Horos (see above). All data used for evaluation are summarized in Table 1.

### Histological assessment of primary and engrafted tumors

Proportions of the primary human tumors were analyzed histologically to assess pathomorphological appearance for comparison with induced xenografts after transplantation. Hematoxylin and Eosin (H&E) stained instantaneous sections of native tumor tissue or formalin fixed and paraffin embedded tissue were utilized for this purpose.

Processing of PDX mice (n = 27) for histological evaluation was performed as described elsewhere [19–21]. Following the MRI, the xenotransplanted animals were anesthetized with isofluorane and sacrificed by decapitation. The brains were dissected and fixed in 4% paraformaldehyde for further histological analyses. H&E staining of paraffin-embedded 3μm serial sections of the mice brains were reviewed histologically by two independent researchers (RB and AH) to determine the percentage of xenograft vitality relative to the overall lesion (Table 1). In this context, regressive changes e.g., calcification, wet keratin or monomorphic fibrosis were defined as non-vital parts. Tumors exhibiting only a percentage of up to 15% vital proportions were designated as non-vital (n = 11). Accordingly, lesions showing more than 15% vital solid tumor content were defined as vital (n = 16) (Table 1, S2 Table). For visualization, the slides were scanned using the Pannoramic MIDI (3D-Histech, Sysmex Europe) and processed using CaseViewer 2.0 software (3D-Histech, Budapest, Hungary).

### Statistical analysis

CETV were taken at identical time points on T1 weighted images 11 minutes after contrast agent application. The statistical significance of all MRI parameters (CETV, AUC, PE, TTP and ADC) was calculated using the two-tailed Mann Whitney test for unpaired values showing no Gaussian distribution (GraphPad Prism 6 software, La Jolla, USA). P values <0.05 were considered as an indication of a significant difference. Longitudinal assessment of tumor volume (n = 5) was calculated using the paired parametric t-test as Gaussian distribution was confirmed by the Shapiro-Wilk normality test.

## Results

### Growth of engrafted tumors—Comparison of qualitative MRI and histological appearances in human and mice

**Fig 1:**
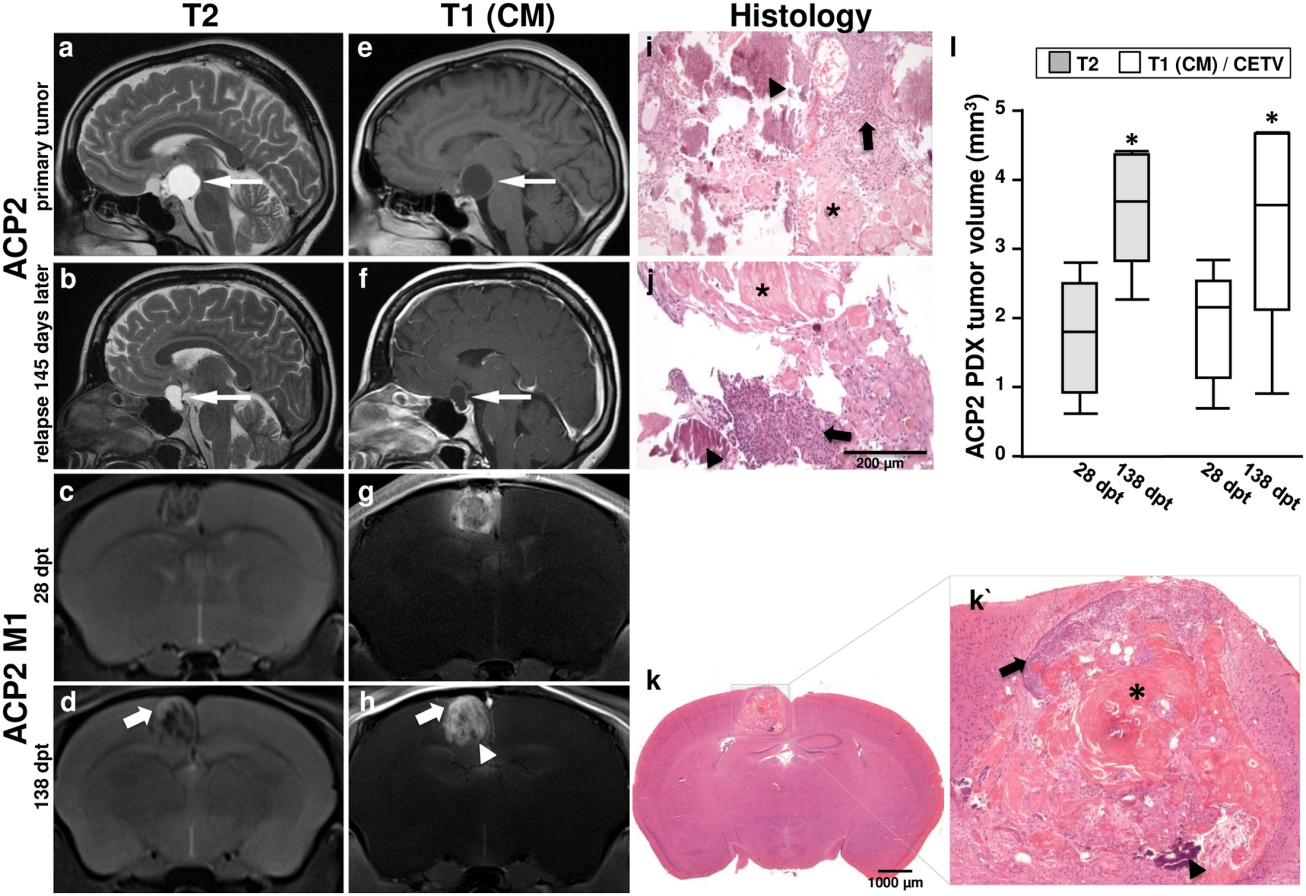
Comparison of primary human MRI and histology with the induced model. MR images and corresponding histology of an ACP patient (ACP2) showing the primary tumor and relapse after 145 days are illustrated in comparison with an example of a direct vital descendent of the surgically removed primary tumor specimen implanted into the nude mouse number one out of ten (ACP2 M1). T2 weighted images show a cystic component within the craniopharyngioma in the human patient (a and b; arrow) with contrast enhancement in the rim of the lesion (e and f; arrow) at baseline (a and e) as compared to 145 days later (b and f) with an increase in size over time. The craniopharyngioma xenograft in the mouse is inhomogeneous in T2 (c and d) and post contrast T1 (g and h) weighted images. The T2w hyperintense and T1 contrast enhancing areas in the tumor increase over 110 days as well (c and d; g and h). Histology of the primary tumor (i) shows similar characteristics to the descendent tumors in human (j) and in mice (k and k`) in terms of vital tumor proportions (arrow), wet keratin (asterisk) and calcification (arrowhead). Vital tumor areas (arrow, k´) in the murine graft were also identified on MRI (arrows, d and h) by T2 hyperintensity and contrast enhancing areas in T1. However, calcified regions appear hypointense (arrowhead, k´, d and h). PDX tumor volume (n = 5) defined 28 and 138 days post transplantation (dpt) in T2 weighted as well as post contrast T1 weighted images revealed a significant increase over time (p<0.05) (l).

Concerning the MRI phenotype of ACP in humans, presurgical evaluation of patients (ACP1-3) revealed lobulated masses with T2 hyperintense cystic and contrast-enhancing solid components of the sellar and suprasellar regions. ACP2 clinically presented a cystic lesion which extends to the suprasellar region (Fig 1a and 1e) compressing adjacent brain structures (Fig 1a and 1e). Over a period of 415 days, a relapse with a cystic sellar mass that enhanced vividly at the rim of the lesion could be detected (Fig 1b and 1f). The resected tissue revealed histologically hallmarks of the ACP subtype in each case including solid tumor tissue (arrow), and wet keratin (asterisk), as well as calcifications (arrowhead) (Fig 1i and 1j).

Orthotopic implantation of tumor material received after the first surgical intervention of ACP2 resulted in inhomogeneous masses in the cortex of mice with T2 iso- to hyperintense MRI appearance (Fig 1c) that avidly enhanced after intravenous contrast media application (Fig 1g), but lacked a clear cystic component. A second MR measurement of PDX after 110 days revealed hypointense foci on T1 and T2 weighted images (Fig 1d and 1h) which correlate histologically with calcifications (Fig 1k and 1k´ arrowhead). These calcifications are also visible in the patient tumor (Fig 1i arrowhead) as well as in the regrown lesion 415 days later (Fig 1j arrowhead). However, regions with vital tumor content in mice appeared hyperintense in T2 weighted images and enhanced in post contrast T1 weighted images (Fig 1d arrow and Fig 1k´ arrow). It is worth noting that the tumor volume of ACP2 PDX (n = 5) increased within 110 days significantly (p<0.05, paired t-test) in relation to the first imaging, as determined on T2 weighted images (median1.80 mm^3^ 28dpt vs. 3.69 mm^3^ 138dpt, p = 0.003) as well as T1 weighted images (median 2.16 mm^3^ 28dpt vs. 3.64 mm^3^ 138dpt, p = 0.027, Fig 1, S1 Table).

### Qualitative histological analysis in correlation with MRI

**Fig 2:**
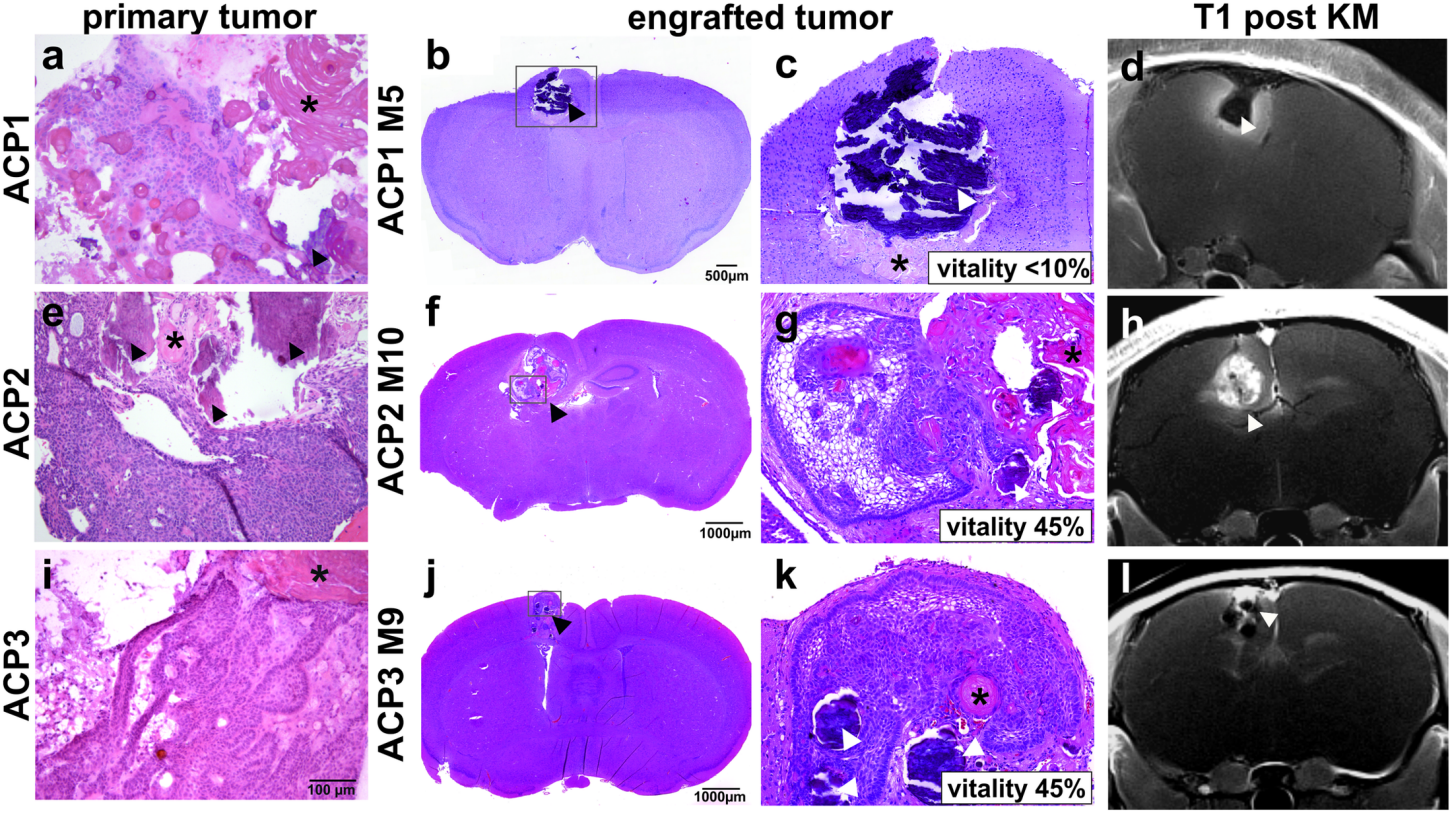
Comparison of patient specific engrafted tumors. Primary (a, e and i) and descended transplanted ACP tissue (b, c, f, g, j and k) show comparable histological appearance with regressive changes e.g. wet keratin (*) and calcifications (arrowhead) with estimated vitality indicated in the magnified images. Associated T1 weighted MR images after contrast medium application (d, h and l) are shown for comparison with histological appearance.

The initial histological nature of the transplanted ACP tumor fragments originating from three different patients (ACP1-3, Table 1) is presented in Fig 2. The tumor of ACP1 (Fig 2a) shows only few vital tumor cells but regressive changes with wet keratin and calcifications predominate. ACP2 (Fig 2e) comprises mostly solid tumor cells surrounded by picket fence-like palisaded cells and parts showing also wet keratin as well as calcifications. ACP3 (Fig 2i) is characterized by solid well-differentiated epithelium partially forming a basal layer with interspersed wet keratin. The histological appearance of arising tumors in PDX models are shown exemplary for engrafted tumors from each patient e.g. mouse 5 of transplanted ACP1 (ACP1 M5, Fig 2b and 2c), ACP2 M10 (Fig 2f and 2g) and ACP3 M9 (Fig 2j and 2k). The grafts exhibit typical histological features of ACP which are morphologically similar to the corresponding patients tumor including different amounts of vital solid epithelial tumor proportions and non-vital parts e.g. wet keratin (asterisk) as well as calcifications (arrowhead). It is worth noting that dependent of the donor tissue heterogeneity, PDX reveal different percentages of vital epithelial tumor cell content (vitality) in mice ranging between <10%–<15% (ACP1M1-7), 18%–70% (ACP2M1-10) and <10%–55% (ACP3M1-10), respectively (Table 1). In comparison with MRI, contrast vivid proportions were correlated to vital tumor areas (Fig 2g, 2h, 2k and 2l), while in tumors with a vitality of less than 10%, no contrast agent accumulated in the xenotransplanted tumor (Fig 2c and 2d). Therefore, hypointense areas on post contrast T1 weighted images (Fig 2d, 2h and 2l; arrowheads) correlate with calcifications (arrowheads) as well as wet keratin (asterisks; Fig 2c, 2g and 2k).

### Quantitative MRI analysis

**Fig 3:**
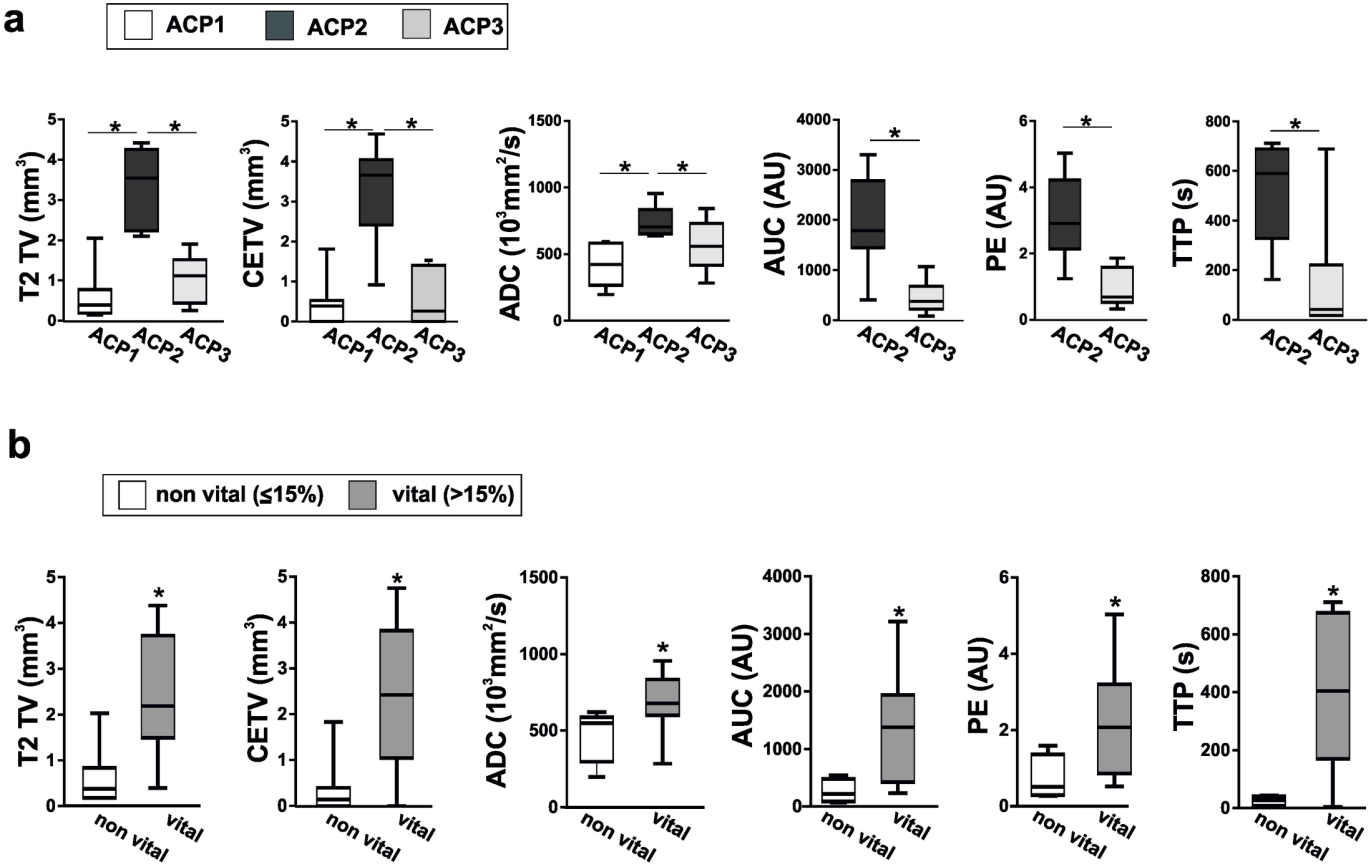
MRI data correlated with patient specific grafts and vitality. a: Patient derived engrafted tumors were analyzed in terms of T2 tumor volume (T2 TV), contrast-enhancing tumor volume (CETV), apparent diffusion coefficient (ADC), area-under-the-curve (AUC), peak enhancement (PE) and time-to-peak (TTP). b: Corresponding data were also evaluated in matters of vitality with a cutoff of 15% discriminating non-vital from vital engrafted tumors. Significant differences are marked by an asterisk (*). Detailed results e.g. mean values and p-values are listed in S2 Table.

On the whole, the tumor take rate of implanted tumor tissue was 100% in mice (n = 27) as diagnosed with MRI. However, quantitative MRI analysis revealed donor-specific differences in the engrafted mice as shown in Fig 3a.

In concordance with the histologically assessed tumor vitality, varying on average between <10% (ACP1, n = 7), ~41% (ACP2, n = 10) and ~26% (ACP3, n = 10), the T2 volume of ACP2 differs significantly from ACP1 and ACP3. Similarly, the evaluation of quantitative MRI data (Fig 3a) revealed a significant enhancement in ACP2 xenografts regarding the volume of contrast-enhancing areas (CETV) and ADC in relation to ACP1 and ACP3. Since parameters of vascularization (AUC, PE and TTP) were not measurable in ACP1, only ACP2 and ACP3 could be compared in this regard. AUC, PE and TTP were significantly increased in ACP2 in comparison to ACP3. The data of all measurements are summarized in Table 1 and respective results including median and p values of all analyses are shown in S2 Table.

In order to evaluate MRI based differentiation of histologically vital and non-vital tumors, we compared all mice showing a vitality ≤15% (n = 11) in the orthotopically implanted tumors with those presenting a higher percentage in vital tumor cells of >15% (n = 16; Table 1; Fig 3b). Using this cut off value of 15% for vitality, all MRI parameters e.g. T2 TV, CETV, ADC, AUC, PE and TTP were significantly higher in vital tumors (Fig 3b).

## Discussion

ACP are epithelial sellar brain tumors that originate from embryonic epithelial cells. It is generally assumed that they arise from remnants of Rathke’s pouch [7]. Despite their benign nature, the removal of ACP still presents a challenge due to their localization within the brain near the hypothalamus, pituitary stalk, optic chiasm and carotid arteries.

Although CT and MRI is the current standard of care to assess craniopharyngiomas by imaging, there are few reports dealing with animal models and non-invasive imaging. These either describe the effects of craniopharyngioma-fluid on rats [23, 24] or the development of spontaneous craniopharyngiomas [25, 26] in rodents. It is worth noting that transgenic ACP mouse models were shown to recapitulate MRI features of human ACP, making it to an important completion of PDX for studying novel therapeutic treatment options [17]. However, only one publication deals with the generation of a xenograft model via subcutaneous tumor cell injection [27]. Neither were organotypic implantations used, nor was MRI included in this report. Therefore, this study is the first description of the MRI properties in an organotypic ACP PDX model.

In clinical MRI, craniopharyngiomas frequently present with a combination of solid tumor tissue, calcifications and cyst formation [28, 29]. In contrast to the clinical situation, we observed a rather solid growth pattern in the mouse model without cystic structures, which might be explained by the fact that primarily the solid tumor parts were xenografted in mice. In concordance with the clinical presentation, hypointense foci in the xenografted tumors were shown to correlate with calcifications. The histological appearance also resembled the human presentation. Histologically determined proportions of wet keratin and calcification could be localized on MR images presenting as hypointense foci on T1 and T2 weighted imaging.

When observing the xenotransplanted tumors longitudinally, post-contrast T1 and T2 weighted imaging indicated that the contrast enhancing (T1) and hyperintense (T2) tumor parts increased significantly in ACP2 engrafted tumors after 110 days. This result is concordant with the clinical course of ACP2 patient revealing tumor regrowth after resection as well. Notwithstanding that craniopharyngiomas are generally considered to be slow-growing tumors [30], we were able to confirm an increase of tumor volume in xenografts within this limited time frame. The proof of the growth of solid tumor compartments underscores this PDX model as a suitable tool for future testing of craniopharyngioma treatment modes and revealed assessment of tumor volume as an appropriate parameter.

Beyond that, the biggest challenge for future treatment experiments is discriminating between vital and non-vital tumor parts *in vivo*. In this study we have shown how to accomplish that by means of MRI. T2 hyperintense and contrast enhancing tumor volumes were shown to be the most appropriate MRI parameters. Therefore, the T2TV and CETV could discriminate between ACP2 with a mean vitality of 41% from ACP 1 and 3 with mean vitalities of <10% and 26%, respectively. Furthermore, we distinguished vital from non-vital tumors with a cut-off value of 15% vitality, hereby T2TV and CETV were significantly higher in the vital tumors.

In addition to morphologic imaging, we employed the functional MRI techniques DWI and DCE-MRI that are abundantly used in clinical routine imaging. The apparent diffusion coefficient (ADC) from DWI plays an important role in diagnosis [31–35] and grading [36–38] of intracranial tumors. It was observed that ADC values help to differentiate craniopharyngiomas from germ cell tumors and normal cerebellar parenchyma in patients [31, 39]. DCE-MRI parameters AUC, PE and TTP are associated with vascularization, which are widely used in tumor diagnosis [40] and in follow-up imaging, for example, to predict response to chemotherapy [41]. PE has, for example, proven valuable for detecting and diagnosing intracranial tumors [39] and for predicting survival after induction chemotherapy [36] in patients suffering from leukemia and to distinguish between germ cell tumors and craniopharyngiomas [31].

In our study, ADC values were significantly higher in vital tumors when compared to non-vital PDX. This accords with significantly increased ADC values in ACP2 derived xenografts relative to ACP1 and ACP3 PDX, and higher average vital tumor content. Thereby, the ADC reflects the increased Brownian motion of water molecules in vital tumors while the water diffusion is most likely suppressed in less vital tumors due to the presence of regressive changes such as wet keratin and calcifications. Accordingly, the DCE-MRI parameters AUC and PE associated with blood volume were significantly higher in vital compared to non-vital tumors. In concordance with this, Jung and colleagues reported that AUC was shown to be a good discriminator between hyper- and hypovascular cerebral neoplasms [42]. Finally, the TTP was also significantly higher in vital than in non-vital xenografts, which might result from a more tortuous vasculature in vital tumors, reducing the wash-in of the contrast agent. As reported above for ADC values, the explanation for a reduced vascularization in non-vital tumors might again be the finding that histological assessment revealed a higher grade of calcifications and non-vital wet keratin in these xenografts.

## Conclusions

This study describes for the first time the MRI properties of an organotypic craniopharyngioma model. Xenotransplanted ACP tumors are slowly growing, as in primary tumors. Vital tumor parts could be assessed with T2 and post contrast T1 weighted images. Furthermore, among non-invasive functional imaging techniques, DWI and DCE-MRI helped to differentiate vital from non-vital xenografts *in vivo*. This achievement is utterly important in regard of preclinical studies to determine treatment response using novel therapeutic strategies.

## Supporting information

S1 Table Analysis of tumor growth. CETV (mm^3^) and T2-TV (mm^3^) data of a longitudinal ACP2 PDX (M1-M5) measurement 28 days post transplantation (dpt) and 138 dpt are listed. Furthermore the median and mean values of CETV (mm^3^) and T2-TV (mm^3^) are shown including statistical significant results (p<0.05). The p-values were determined using the parametric paired t-test.
(DOCX)

S2 Table Supplemental MRI data. Results of MRI data including median values, the 25% as well as 75% percentile, and the p-values of xenotransplanted models grouped respectively according to primary tumor specific origin or vitality. The amount of enclosed animals in each group (n) is also displayed. The p-values were determined using the Mann Whitney test and significant results are printed in bold type.
(DOCX)
