# Supplementary material for: Characterization of the murine orthotopic adamantinomatous craniopharyngioma PDX model by MRI in correlation with histology
Source: PLoS One. 2018 May 24;13(5):e0197895. doi: 10.1371/journal.pone.0197895 (PMC5993109; doi:10.1371/journal.pone.0197895)
Supplement: S1 Table — CETV (mm3) and T2-TV (mm3) data of a longitudinal ACP2 PDX (M1-M5) measurement 28 days post transplantation (dpt) and 138 dpt are listed. Furthermore the median and mean values of CETV (mm3) and T2-TV (mm3) are shown including statistical significant results (p<0.05). The p-values were determined using the parametric paired t-test. (DOCX) [file pone.0197895.s001.docx]

Supplemental Table 1

| **Longitudinal evaluation of ACP2 PDX** | | | | | |
| --- | --- | --- | --- | --- | --- |
| **CETV (mm^3^)** | | | **T2-TV (mm^3^)** | | |
| ***PDX*** | ***28 dpt*** | ***138 dpt*** | ***PDX*** | ***28 dpt*** | ***138 dpt*** |
| M1 | 2.848 | 3.639 | M1 | 2.803 | 3.69 |
| M2 | 0.699 | 0.915 | M2 | 0.617 | 2.267 |
| M3 | 1.586 | 3.335 | M3 | 1.238 | 3.397 |
| M4 | 2.237 | 4.689 | M4 | 2.213 | 4.319 |
| M5 | 2.160 | 4.662 | M5 | 1.801 | 4.419 |
| **median** | **2.16** | **3.639** | **median** | **1.801** | **3.690** |
| **mean** | **1.906** | **3.448** | **mean** | **1.734** | **3.618** |
| **Paired t-test**  **p-value** | **0.027** | | **Paired t-test**  **p-value** | **0.003** | |
