# Supplementary material for: Characterization of the murine orthotopic adamantinomatous craniopharyngioma PDX model by MRI in correlation with histology
Source: PLoS One. 2018 May 24;13(5):e0197895. doi: 10.1371/journal.pone.0197895 (PMC5993109; doi:10.1371/journal.pone.0197895)
Supplement: S2 Table — Results of MRI data including median values, the 25% as well as 75% percentile, and the p-values of xenotransplanted models grouped respectively according to primary tumor specific origin or vitality. The amount of enclosed animals in each group (n) is also displayed. The p-values were determined using the Mann Whitney test and significant results are printed in bold type. (DOCX) [file pone.0197895.s002.docx]

|  | **Patient specific evaluation** | | | | | | | | | | | | |
| --- | --- | --- | --- | --- | --- | --- | --- | --- | --- | --- | --- | --- | --- |
|  | **ACP1** | | | **ACP2** | | | **ACP3** | | | | **ACP1 vs. ACP2** | **ACP1 vs. ACP3** | **ACP2 vs. ACP3** |
|  | *n* | *median* | *25%/75% Percentile* | *n* | *median* | *25%/75% Percentile* | *n* | *median* | | *25%/75% Percentile* | *p-value* | *p-value* | *p-value* |
| **T2-TV (mm^3^)** | 7 | 0.389 | 0.1889/0.775 | 10 | 3.544 | 2.239/4.258 | 8 | 1.120 | | 0.4445/1.518 | **0.0001** | 0.0939 | **<0.0001** |
| **CETV (mm^3^)** | 7 | 0.391 | 0/0.528 | 10 | 3.659 | 2.414/4.047 | 10 | 0.2585 | | 0/1.401 | **0.0002** | 0.9614 | **<0.0001** |
| **ADC (10^3^mm^2^/s)** | 6 | 423.4 | 266.9/583.2 | 10 | 704.5 | 652.8/834.7 | 9 | 559.2 | | 418/843.6 | **0.0002** | 0.2238 | **0.0172** |
| **AUC** | nap | nap | nap | 10 | 1762 | 1427/2734 | 10 | 381.1 | | 232.6/674.4 | nap | nap | **0.0002** |
| **PE** | nap | nap | nap | 10 | 2.91 | 2.14/4.219 | 10 | 0.6885 | | 0.531/1.583 | nap | nap | **<0.0001** |
| **TTP (s)** | nap | nap | nap | 10 | 590.6 | 328.3/688.7 | 10 | 42.04 | | 18.26/220.7 | nap | nap | **0.0016** |
|  | **Vitality specific evaluation** | | | | | | | |  |  |  |  |  |
|  | **Non vital (≤15% vitality)** | | | **vital (>15% vitality)** | | | **Vital vs. non vital** | |  |  |  |  |  |
|  | *n* | *median* | *25%/75% Percentile* | *n* | *median* | *25%/75% Percentile* | *p-value* | |  |  |  |  |  |
| **T2-TV (mm^3^)** | 9 | 0.389 | 0.189/0.852 | 16 | 2.211 | 1.513/3.76 | **0.0001** | |  |  |  |  |  |
| **CETV (mm^3^)** | 11 | 0.138 | 0/0.394 | 16 | 2.393 | 1.034/3.774 | **0.0006** | |  |  |  |  |  |
| **ADC (10^3^mm^2^/s)** | 9 | 547.6 | 297/586.4 | 16 | 677.6 | 600.4/834.7 | **0.0035** | |  |  |  |  |  |
| **AUC** | 4 | 240.2 | 111.3/506.1 | 16 | 1406 | 451/1966 | **0.0157** | |  |  |  |  |  |
| **PE** | 4 | 0.561 | 0.346/1.403 | 16 | 2.106 | 0.911/3.224 | **0.0293** | |  |  |  |  |  |
| **TTP (s)** | 4 | 30.02 | 10.75/44.04 | 16 | 406.4 | 173.2/674.9 | **0.0080** | |  |  |  |  |  |
